# A subcutaneous nodule in a returning Chinese expatriate

**DOI:** 10.1371/journal.pntd.0007073

**Published:** 2019-08-22

**Authors:** Xin-yu Wang, Qiao-ling Ruan, Peng Cui, Wen-hong Zhang

**Affiliations:** Department of Infectious Diseases, Huashan Hospital, Fusan University, Shanghai, China; Vienna, AUSTRIA

## Case discussion and question

**Fig 1:**
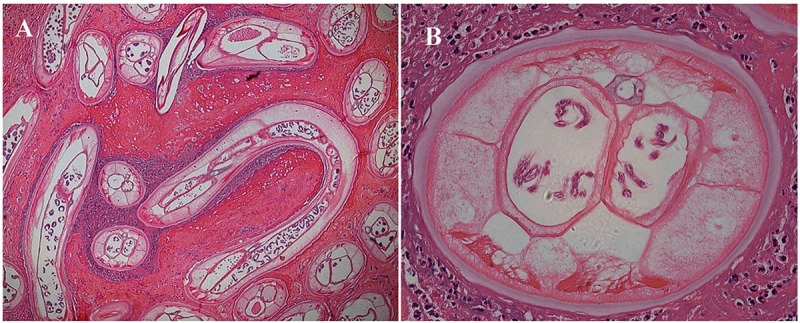
Histopathological examination of the lump after biopsy. Panel A. Hematoxylin and eosin staining shows several adult worms (original magnification ×40). Panel B. Transverse section of a worm (original magnification ×400).

A 51-year-old Chinese man presented with a lump on his left buttock. Two years prior, he worked as a gold dredging contractor for one year in Banalia, Tshopo province, the Democratic Republic of the Congo, and received repeated unidentified insect bites. Three months prior, he found a bean-sized lump on his left buttock. He then developed an erythematous itchy rash on his legs that extended over his whole body, without fever or enlarged lymph nodes. The rash improved without treatment. The lump enlarged gradually over two months. On physical examination, the lump (diameter 2.5 cm) was not tender or movable. The patient was afebrile and did not have palpable inguinal lymphadenopathy. Laboratory data revealed normal neutrophils and eosinophils, C-reactive protein level, and erythrocyte sedimentation rate. Ultrasound revealed a low echogenic subcutaneous area (27 × 15 × 18 mm) and subcutaneous edema. Excisional biopsy and histopathological examination were performed (Fig 1). What is your diagnosis?

## Answer and discussion

### Diagnosis: *Onchocerca volvulus* infection (onchocercoma)

**Fig 2:**
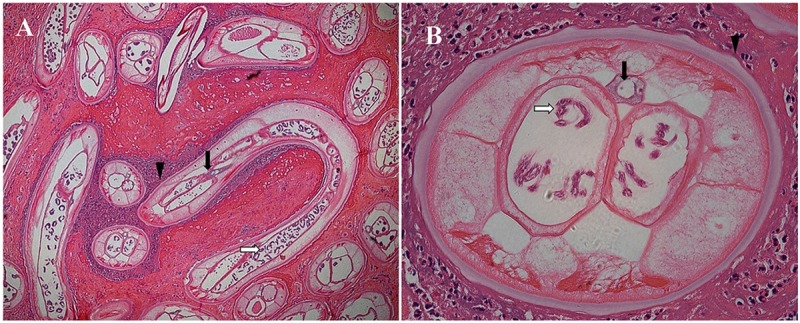
Histopathological examination of the lump after biopsy. Panel A. Hematoxylin and eosin staining shows several adult *O*. *volvulus* worms, including an intact longitudinal section of an adult female worm (original magnification ×40). Panel B. Transverse section of a female *O*. *volvulus* worm surrounded by inflammatory cells (original magnification ×400). The key diagnostic features include the cuticular rings (darts), paired uterine branches containing coiled microfilaria (white arrows), and the intestine (black arrows).

The microscopic findings of the excisional biopsy were diagnostic of *O*. *volvulus* (Fig 2). Hematoxylin and eosin staining showed several adult *O*. *volvulus* worms surrounded by a fibrous capsule. The pivotal diagnostic characteristics included the cuticular rings, paired uterine branches containing coiled microfilaria, and the intestine. Moreover, conventional polymerase chain reaction was used to amplify an *O*. *volvulus*–specific repeat DNA sequence, which confirmed the diagnosis. Additionally, the patient had a thorough ophthalmologic examination to detect microfilariae, and no involvement was noted. Specifically, there were no microfilariae in the blood, bone marrow, or cerebrospinal fluid.

With increasing international travel and the exportation of labor service, there is rising exposure to pathogens that would infrequently be encountered in nonendemic countries. Imported tropical diseases may present with nonspecific symptoms years after exposure, posing a difficult diagnostic dilemma for clinicians in nonendemic areas. In mainland China, malaria and dengue are the most commonly diagnosed imported diseases among travelers from Africa [1]. Onchocerciasis is endemic to much of sub-Saharan Africa. Small endemic foci are also present in the Arabian Peninsula (Yemen) and Americas (Brazil and Venezuela). Onchocerciasis has been rarely reported as an imported illness in recent years due to its successful control [2]. Thus, its clinical presentation is usually underappreciated. Moreover, the clinical presentation of filarioid disease is known to differ between visitors to and natives of endemic regions. Those born in regions where *O*. *volvulus* infection is endemic generally have a lower possibility of skin microfilariae and less ocular disease than do visitors to these regions [3]. Travel history is of great clinical importance. Expatriate groups are highly susceptible because of their relatively long-term exposure in endemic countries. However, there are several cases of *O*. *volvulus* infection after short stays (2–6 weeks) [2].

*O*. *volvulus* is transmitted by *Simulium* spp. blackflies, and infective larvae are incubated during the bite. The larvae mature to female and male worms within one year and are located in subcutaneous nodules (onchocercomata) [4]. The long-living females produce millions of microfilariae (first-stage larvae) during their lifetime, potentially causing dermatitis, lymphadenitis, and ocular lesions, which can progress to visual impairment and blindness [5]. Clinical manifestations may develop months or years later and vary according to the parasite intensity. Our patient had a long incubation period. Slightly infected individuals may remain asymptomatic with eosinophilia or elevated immunoglobulin E. Symptoms in travelers are primarily dermatologic in nature and may occur years after departure from endemic areas. The most prevalent cutaneous manifestation is a highly pruritic papular lesion, whereas more chronic changes can include atrophy, hyperkeratosis, and abnormal pigmentation [4]. Nodules are more common in endemic populations. Onchocercomata most frequently occur on the pelvis [4], as observed in our patient.

A clinical suspicion of onchocerciasis should be evoked when travelers from endemic regions have skin lesions compatible with the disease. However, the differential diagnoses should include a variety of parasitic infections (e.g., loiasis, *Mansonella* infection, African trypanosomiasis, schistosomiasis, and cutaneous leishmaniasis). The characteristic skin lesions of loiasis are transient localized subcutaneous swellings (Calabar swellings) on the face and extremities. Coinfection with loiasis should be carefully ruled out. Cutaneous leishmaniasis lesions can be initially nodular and then develop into diffuse nodular eruptions. Disseminated fungal infections can also cause nodular lesions. A diagnosis of onchocerciasis is usually based on the presence of microfilariae in superficial skin shavings or a punch biopsy, with adult worms in histologic sections of excised nodules. Being able to recognize the key morphologic characteristics of adult *O*. *volvulus* worms under the microscope favors the diagnosis. However, a diagnosis is often difficult in nonendemic areas because young female worms do not yet produce microfilariae, which are definitively diagnostic for onchocerciasis when detected in the skin [6]. Microfilariae can be detected, although not frequently, in the peripheral blood of patients with high parasitemia. Serologic testing is helpful for detecting the infection; however, the tests may not be available in nonendemic countries, such as China. Additionally, molecular tests are useful tools for confirming the diagnosis.

The primary treatment for onchocerciasis is ivermectin, which kills the microfilariae but not the adult worms. As *O*. *volvulus* is associated with the essential endosymbiotic bacterium *Wolbachia*, anti-*Wolbachia* therapies (including doxycycline) are an evolving treatment strategy for onchocerciasis [4]. Some experts recommend treating patients with one dose of ivermectin followed by six weeks of doxycycline. After a thorough evaluation and nodule excision, our patient remained symptom-free under close follow-up for one year without ivermectin. Thus, we wonder if patients with a single nodule who undergo nodulectomy need further antiparasitic treatment.

In summary, this is a typical case of imported onchocerciasis, which can be easily missed by clinicians in nonendemic areas. Most infections in nonendemic areas occur in expatriate groups and may present years after departure from the endemic area. A single subcutaneous nodule may indicate the disease; however, a differential diagnosis is necessary. Typical microscopic findings and molecular identification can be used to confirm the diagnosis.

**Key learning points**

- *O*. *volvulus* infection can be missed by clinicians in nonendemic areas.
- The clinical presentation of imported *O*. *volvulus* infection may differ from that in natives of endemic areas.
- Biopsy and typical microscopic findings can aid in the diagnosis of onchocercoma.

### Ethics statements

The patient in this manuscript provided written informed consent (as outlined in the PLoS consent form) to the publication of their case details.
